# CpG Dinucleotides Inhibit HIV-1 Replication through Zinc Finger Antiviral Protein (ZAP)-Dependent and -Independent Mechanisms

**DOI:** 10.1128/JVI.01337-19

**Published:** 2020-02-28

**Authors:** Mattia Ficarelli, Irati Antzin-Anduetza, Rupert Hugh-White, Andrew E. Firth, Helin Sertkaya, Harry Wilson, Stuart J. D. Neil, Reiner Schulz, Chad M. Swanson

**Affiliations:** a Department of Infectious Diseases, King’s College London, London, United Kingdom; b Department of Medical and Molecular Genetics, King’s College London, London, United Kingdom; c Division of Virology, University of Cambridge, Cambridge, United Kingdom; Icahn School of Medicine at Mount Sinai

**Keywords:** CpG, KHNYN, RNA, ZAP, human immunodeficiency virus, innate immunity, virus

## Abstract

CpG dinucleotides are suppressed in the genomes of many vertebrate RNA viruses, including HIV-1. The cellular antiviral protein ZAP (zinc finger antiviral protein) binds CpGs and inhibits HIV-1 replication when CpGs are introduced into the viral genome. However, it is not known if ZAP-mediated restriction is the only mechanism driving CpG suppression. To determine how CpG dinucleotides affect HIV-1 replication, we increased their abundance in multiple regions of the viral genome and analyzed the effect on RNA expression, protein abundance, and infectious-virus production. We found that the antiviral effect of CpGs was not correlated with their abundance. Interestingly, CpGs inserted into some regions of the genome sensitize the virus to ZAP antiviral activity more efficiently than insertions into other regions, and this sensitivity can be modulated by interferon treatment or ZAP overexpression. Furthermore, the sensitivity of the virus to endogenous ZAP was correlated with its sensitivity to the ZAP cofactor KHNYN. Finally, we show that CpGs in some contexts can also inhibit HIV-1 replication by ZAP-independent mechanisms, and one of these is the activation of a cryptic splice site at the expense of a canonical splice site. Overall, we show that the location and sequence context of the CpG in the viral genome determines its antiviral activity.

**IMPORTANCE** Some RNA virus genomes are suppressed in the nucleotide combination of a cytosine followed by a guanosine (CpG), indicating that they are detrimental to the virus. The antiviral protein ZAP binds viral RNA containing CpGs and prevents the virus from multiplying. However, it remains unknown how the number and position of CpGs in viral genomes affect restriction by ZAP and whether CpGs have other antiviral mechanisms. Importantly, manipulating the CpG content in viral genomes could help create new vaccines. HIV-1 shows marked CpG suppression, and by introducing CpGs into its genome, we show that ZAP efficiently targets a specific region of the viral genome, that the number of CpGs does not predict the magnitude of antiviral activity, and that CpGs can inhibit HIV-1 gene expression through a ZAP-independent mechanism. Overall, the position of CpGs in the HIV-1 genome determines the magnitude and mechanism through which they inhibit the virus.

## Abstract (precis)

Some RNA virus genomes are suppressed in the nucleotide combination of a cytosine followed by a guanosine (CpG), indicating that they are detrimental to the virus. The antiviral protein ZAP binds viral RNA containing CpGs and prevents the virus from multiplying. However, it remains unknown how the number and position of CpGs in viral genomes affect restriction by ZAP and whether CpGs have other antiviral mechanisms. Importantly, manipulating the CpG content in viral genomes could help create new vaccines. HIV-1 shows marked CpG suppression, and by introducing CpGs into its genome, we show that ZAP efficiently targets a specific region of the viral genome, that the number of CpGs does not predict the magnitude of antiviral activity, and that CpGs can inhibit HIV-1 gene expression through a ZAP-independent mechanism. Overall, the position of CpGs in the HIV-1 genome determines the magnitude and mechanism through which they inhibit the virus.

## INTRODUCTION

The frequency of CpG dinucleotides is suppressed in many vertebrate RNA viruses, indicating that they may be deleterious (1–5). Supporting this hypothesis, increasing the CpG content in picornaviruses and influenza A virus inhibits their replication (6–10). However, the mechanisms by which CpG dinucleotides attenuate viral replication remain unclear. Importantly, introduction of CpG dinucleotides into viral genomes using synthetic biology techniques may be a new way to develop live attenuated virus vaccines, and a full understanding of how CpG dinucleotides inhibit viral replication is necessary to develop this approach (9, 10).

HIV-1 encodes the three polyproteins found in all retroviruses (Gag, Pol, and Env), two regulatory proteins (Tat and Rev), and four accessory proteins (Vif, Vpr, Vpu, and Nef) (11). CpG dinucleotides are suppressed throughout the HIV-1 genomic RNA (gRNA), and introducing CpGs into *gag* or *env* inhibits viral replication (12–16). Furthermore, analysis of clinical HIV-1 samples found that mutations that create new CpG dinucleotides in HIV-1 are twice as costly as those that do not and that increased CpG dinucleotide abundance in *env* may predict disease progression (17, 18). There are at least four mechanisms by which CpG dinucleotides could inhibit HIV-1. First, CpG DNA methylation-induced transcriptional silencing could repress viral gene expression (12, 13, 19). Second, introduction of CpGs into *cis*-acting elements or structures required for viral replication may render them nonfunctional. Third, CpGs could create deleterious *cis*-acting elements or structures. Fourth, they could act as a pathogen-associated molecular pattern (PAMP) that is recognized by the innate immune system. Supporting the hypothesis that CpGs in viral RNA could be a PAMP, the antiviral protein ZAP (zinc finger antiviral protein) has recently been shown to bind regions of HIV-1 RNA containing CpG dinucleotides and to inhibit HIV-1 with increased CpG content in *env* (16).

ZAP (encoded by the gene *ZC3HAV1*) was initially discovered as a cellular factor inhibiting murine leukemia virus (MLV) gene expression (20). Subsequent studies have shown that ZAP also inhibits alphaviruses, filoviruses, and hepadnaviruses, as well as some flaviviruses and picornaviruses (21–25). ZAP inhibits viral replication by binding viral RNA and targeting it for degradation and/or inhibiting its translation (20, 21, 26–28). It may also have other mechanisms of antiviral activity. However, ZAP does not restrict all viruses, and yellow fever virus, Zika virus, dengue virus, herpes simplex virus 1, vesicular stomatitis virus, and poliovirus are resistant to its antiviral activity (21, 24). ZAP does not have enzymatic activity and interacts with other cellular proteins, such as TRIM25 and KHNYN, to restrict viral replication (29–31). Why some viruses are sensitive to ZAP and others are resistant, and whether the CpG abundance and context in viruses determines this, remains unknown. It is also unclear whether the deleterious effect of CpG dinucleotides on viral replication is mediated exclusively by ZAP or through additional mechanisms.

Overall, the specific mechanisms by which CpG dinucleotides inhibit viral replication are not well understood for any virus. Because CpGs are highly suppressed in HIV-1, it is an excellent model virus to study the antiviral effects of the dinucleotide. In this study, we introduced CpGs into different contexts and regions of the viral genome and analyzed how they restricted viral gene expression and replication. First, we determined whether there was a position-dependent effect of CpGs on viral replication. Second, we analyzed whether there was a correlation between endogenous ZAP activity and the abundance of CpG dinucleotides. Third, we tested whether increasing ZAP abundance increased its antiviral effect. In sum, we found that CpGs in different contexts and locations inhibited viral gene expression through ZAP-dependent inhibition of gene expression and ZAP-independent changes in pre-mRNA splicing. Importantly, the number of introduced CpG dinucleotides did not predict the magnitude of their antiviral activity or inhibition by endogenous ZAP. ZAP appears to target a specific region in *env* containing introduced CpGs more efficiently than other regions in the viral genome, though high levels of ZAP can target most regions of the genome containing CpGs. Our results indicate that the context and position of CpG dinucleotides in the HIV-1 genome determine how they inhibit viral replication through ZAP-dependent and -independent mechanisms.

## RESULTS

**FIG 1:**
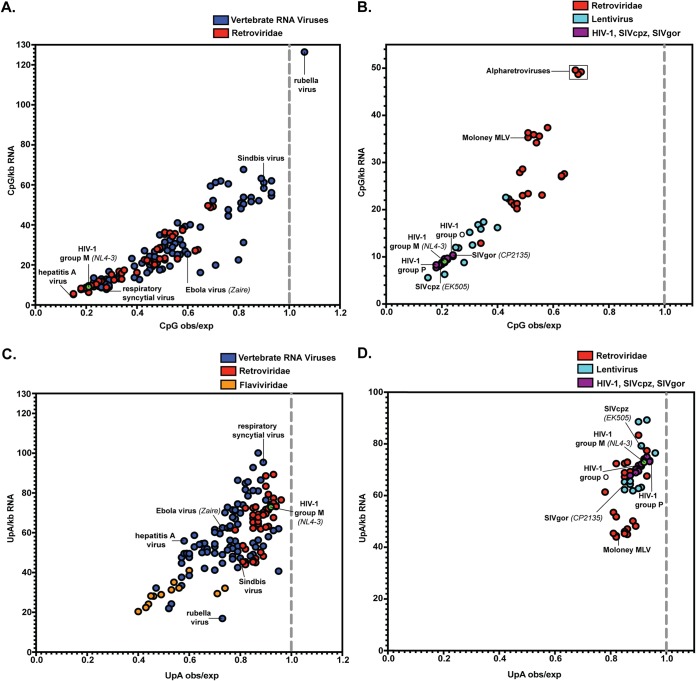
CpG abundance is highly suppressed and UpA abundance is not substantially suppressed in HIV-1 compared to many viruses that infect vertebrate cells. (A) The number of CpGs per kilobase and the CpG observed (obs)/expected (exp) ratio were calculated for each virus. (B) The numbers of CpGs per kilobase and CpG observed/expected ratio were plotted for the family *Retroviridae*. Members of the genus *Lentivirus* and the other retroviruses are also shown. The viruses naturally found in humans, chimpanzees, and gorillas are shown in purple. (C) The number of UpAs per kilobase and the UpA observed/expected ratio were calculated for each virus. (D) The numbers of UpAs per kilobase and UpA observed/expected ratio were plotted for the family *Retroviridae*. The viruses naturally found in humans, chimpanzees, and gorillas are shown in purple. See Data Set S1 for the value for each virus.

To determine how CpG suppression in HIV-1 compared with that in other viruses that infect vertebrates, we compared the CpG frequencies in a panel of viruses (Fig. 1A; see Data Set S1 in the supplemental material). Because RNA viruses have large variations in the frequencies of A, C, G, and U in their genomes, we calculated the number of CpGs per kilobase of RNA and the observed/expected ratio of CpGs. This analysis showed that there is a broad range of CpG suppression in RNA viruses. As previously shown, togaviruses show little CpG suppression, with >40 CpGs/kb and an observed/expected ratio of >0.75 (1–5). Many viruses show moderate CpG suppression, with an observed/expected CpG ratio of ∼0.5. However, there are some viruses in which CpG abundance is highly suppressed, including hepatitis A virus, respiratory syncytial virus, and HIV-1 (Fig. 1A; see Data Set S1). Within the retrovirus family, lentiviruses have high levels of CpG suppression (6 to 23 CpGs/kb; observed/expected ratio, 0.2 to 0.4) and alpharetroviruses have low levels of suppression (∼50 CpGs/kb; observed/expected ratio, ∼0.7) (Fig. 1B; see Data Set S1). Viruses closely related to HIV-1 have ∼10 CpGs/kb and an observed/expected ratio of ∼0.2.

In addition to CpG dinucleotides, UpA dinucleotides are suppressed in many RNA viruses (1, 3, 4). Recently, it was reported that ZAP interacts with viral RNA containing UpAs and restricts echovirus 7 containing introduced UpAs (32). Therefore, we analyzed the UpA abundances in our panel of viruses (Fig. 1C; see Data Set S1). While some vertebrate RNA viruses, such as flaviviruses, show marked UpA suppression (<30 UpAs/kb; observed/expected ratio, <0.5) (Fig. 1C), UpA frequency in retroviruses is not substantially suppressed (Fig. 1D). Specifically, viruses closely related to HIV-1 have ∼70 UpAs/kb and observed/expected ratios of ∼0.9. In sum, CpGs, but not UpAs, appear to be potently suppressed in HIV-1.

**FIG 2:**
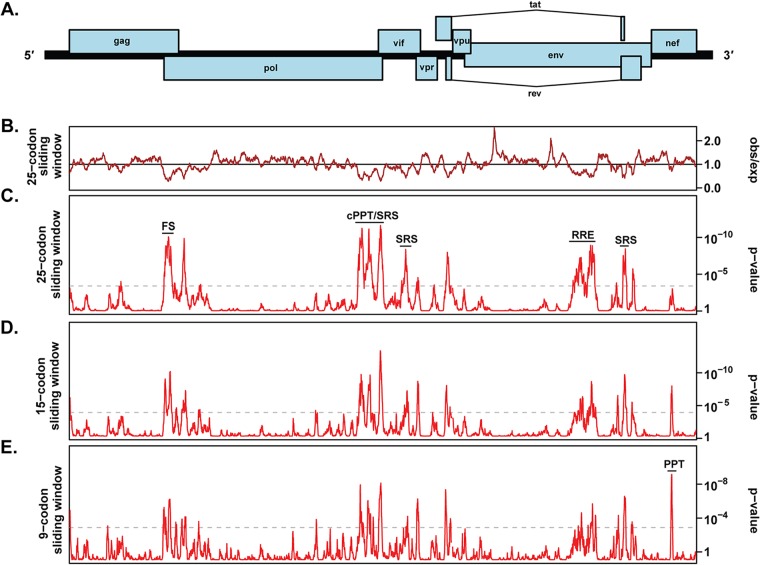
Overlapping functional elements are localized to specific regions of the HIV-1 genome. (A) Schematic representation of the HIV-1 provirus with the open reading frames indicated. (B) Analysis of conservation at synonymous sites. The brown line depicts the ratio of the observed number of substitutions to the number expected under a null model of neutral evolution at synonymous sites for a 25-codon sliding window. (C to E) The red lines show the corresponding *P* values for 25-, 15-, and 9-codon sliding-window analyses. The gray dashed lines indicate approximate false-positive thresholds with a *P* value of 0.05 after correcting for multiple tests in each plot. See Data Set S2 for the z-score for each codon in the HIV-1 open reading frames in the 9-, 15-, or 25-codon sliding windows. The regions with known *cis*-acting elements are identified: FS, programmed ribosomal frameshift sequence (33); cPPT, central polypurine tract (35); SRS, splicing regulatory sequences (36); RRE, Rev response element (34); PPT, polypurine tract (35).

To better understand the potential evolutionary pressures that have led to CpG suppression in HIV-1, we explored the effects of introducing CpGs into different regions of the viral genome using synonymous mutations. However, HIV-1 contains several overlapping reading frames that constrain where CpGs can be introduced (Fig. 2A). Furthermore, it is important that the synonymous mutations introducing CpGs do not disrupt RNA elements that regulate viral replication. The HIV-1 open reading frames (ORFs) contain multiple *cis*-acting regulatory elements, including the programmed ribosomal frameshift sequence in *gag* (33); the Rev response element (RRE) in *env* (34); polypurine tracts in *pol* and *nef* (35); and splicing signals in *pol*, *vif*, *vpr*, *tat*, *rev*, and *env* (36). In addition, there could be uncharacterized elements. Therefore, we identified sequences in the HIV-1 open reading frames that contain reduced variability at synonymous sites, which could indicate the presence of functional RNA elements (37). This analysis identified many of the known HIV-1 linear and structural RNA regulatory elements, including the region at the 5′ end of *gag* required for dimerization and encapsidation, the ribosomal frameshift sequence required for Pol translation, the polypurine tracts required for reverse transcription, several splicing regulatory sequences, and the RRE (Fig. 2B to E; see Data Set S2 in the supplemental material). We synonymously introduced CpG dinucleotides into *gag*, *pol*, and *env* sequences that the analysis revealed were unlikely to contain *cis*-acting elements (see Data Set S2).

### The number of CpGs introduced into the HIV-1 genome is not correlated with the antiviral effect or ZAP sensitivity.

**FIG 3:**
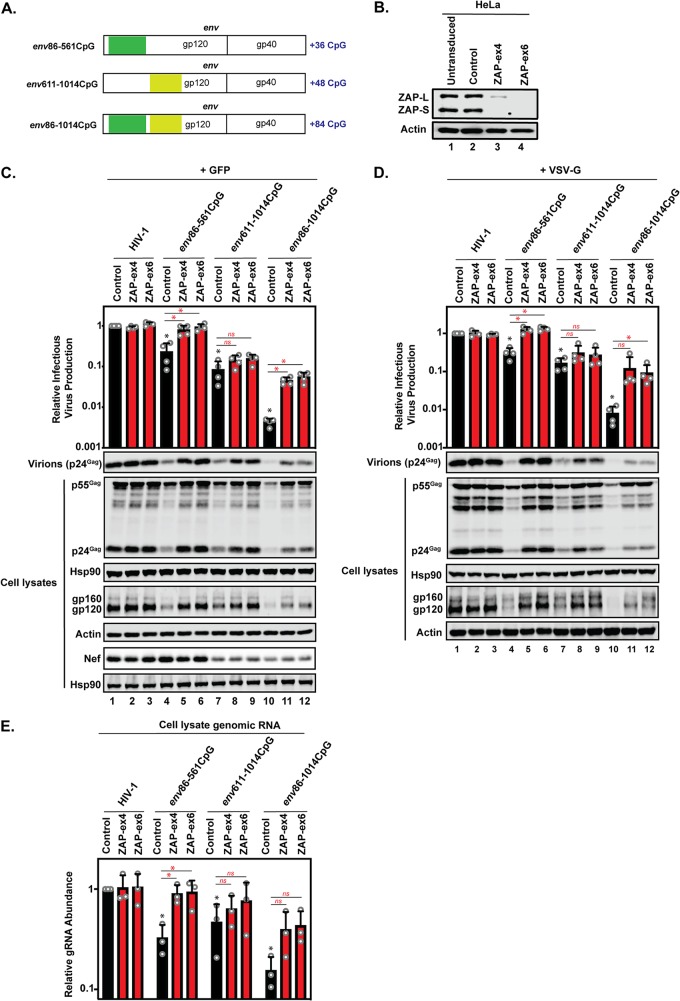
Introduction of CpGs into *env* inhibits HIV-1 infectious-virus production through ZAP-dependent and -independent mechanisms. (A) Schematic representation of the HIV-1 *env* region with the regions of synonymously introduced CpGs in HIV-1*_env_*_86–561_CpG, HIV-1*_env_*_611–1014_CpG, and HIV-1*_env_*_86–1014_CpG highlighted. (B) ZAP expression in HeLa untransduced, control, ZAP-ex4, and ZAP-ex6 CRISPR cells was detected using immunoblotting. The untransduced cells are the parental HeLa cells that were not transduced with a CRISPR-Cas9 lentiviral vector. (C and D) HeLa control, ZAP-ex4, and ZAP-ex6 CRISPR cells were transfected with pHIV-1, pHIV-1*_env_*_86–561_CpG, pHIV-1*_env_*_611–1014_CpG, or pHIV-1*_env_*_86–1014_CpG plus pGFP (C) or pVSV-G (D). The culture supernatants were used to infect TZM-bl reporter cells to measure infectious-virus production. Gag expression in the media, as well as Gag, Hsp90, Env, actin, and Nef expression in the cell lysates, was detected using immunoblotting. The bar charts show the averages of the results of four independent experiments normalized to wild-type HIV-1 in HeLa control CRISPR cells. (E) Genomic-RNA abundance was quantified by qRT-PCR in cell lysates. The bar charts show the averages of the results of three independent experiments normalized to wild-type HIV-1 in HeLa control CRISPR cells. The error bars represent standard deviations. *, *P* < 0.05 as determined by a two-tailed unpaired *t* test; ns, not significant. The black asterisks/ns compare the virus containing introduced CpGs in the control CRISPR cells to wild-type HIV-1 in the control CRISPR cells. The red asterisks/ns compare the virus containing introduced CpGs between the ZAP CRISPR cells (red bars) and the control CRISPR cells (black bars).

An important experimental consideration when studying how CpGs regulate HIV-1 is that CpG DNA methylation-induced transcriptional silencing could potentially inhibit HIV-1 gene expression. However, CpGs in plasmids amplified in bacteria are not methylated when the plasmid is transiently transfected into mammalian cells (38–40), and we have therefore used an experimental approach in which HIV-1 proviral DNA plasmids are transfected into HeLa or 293T cells. A region in *env* immediately after *vpu* does not contain any detectable *cis*-acting RNA elements, which makes it a good region to analyze the effect of introducing CpGs (Fig. 2; see Data Set S2). It has previously been shown that introducing 36 CpGs into *env* nucleotides (nt) 86 to 561 (HIV-1*_env_*_86–561_CpG) inhibits HIV-1 genomic-RNA abundance, Gag expression, Env expression, and infectious-virus production (Fig. 3A, C, and E and Table 1) (16, 31). Importantly, this restriction is eliminated in ZAP knockout cells (Fig. 3B, C, and E), indicating that it is due to the recognition of the CpG dinucleotides by ZAP. To further characterize how CpGs affect HIV-1 replication, we inserted 48 CpGs into *env* nt 611 to 1014 (HIV-1*_env_*_611–1014_CpG) (Fig. 3A and Table 1) and analyzed their effect on HIV-1 genomic-RNA abundance, Env expression, Gag expression, and infectious-virus production. These CpGs inhibited HIV-1 infectious-virus production more potently than the 36 CpGs in HIV-1*_env_*_86–561_CpG in the control CRISPR cells (Fig. 3C). However, in the ZAP knockout cells, Gag expression, Env expression, and virion production were only partially increased for HIV-1*_env_*_611–1014_CpG, and infectious-virus production was rescued only ∼2-fold (Fig. 3C). This indicates that the 48 CpGs introduced into this region of *env* caused both ZAP-dependent and ZAP-independent suppression of infectious-virus production. Nef is expressed from fully spliced mRNAs that do not contain the *env* region with the introduced CpGs (36). As expected, the CpGs introduced into HIV-1*_env_*_86–561_CpG did not decrease Nef expression (Fig. 3C). However, there was decreased Nef expression for HIV-1*_env_*_611–1014_CpG, and this was not rescued in the ZAP CRISPR cells. This suggests that another mechanism, such as altered splicing, contributes to the CpG-mediated decrease in infectious-virus production for HIV-1*_env_*_611–1014_CpG. Furthermore, genomic-RNA abundance for HIV-1*_env_*_611–1014_CpG was not fully restored to wild-type levels in ZAP knockout cells, indicating that the CpGs in HIV-1*_env_*_611–1014_CpG inhibit genomic-RNA abundance through ZAP-dependent and ZAP-independent mechanisms (Fig. 3E).

**TABLE 1:** CpGs and mutations introduced by codon modification

Virus	Total no. within the modified region^*a*^		
	CpGs in wild-type virus	CpGs introduced	Mutations introduced
HIV-1*_gag_*_22–165_CM	4	11	49
HIV-1*_gag_*_22–261_CM	4	18	80
HIV-1*_gag_*_22–378_CM	4	26	109
HIV-1*_gag_*_22–378_CM–no-CpG	4	0	79
HIV-1*_gag_*_22–378_CpG	4	26	30
HIV-1*_gag_*_22–378_DC	4	0	23
HIV-1*_gag_*_22–378_CM–5nt-CpG	4	26	71
HIV-1*_gag_*_22–651_CpG	4	30	37
HIV-1*_gag_*_660–1185_CpG	5	32	36
HIV-1_g_*_ag_*_22–1185_CpG	9	62	73
HIV-1_g_*_ag_*_694–1206_CpG	5	60	72
HIV-1*_pol_*_795–1386_CpG	1	53	61
HIV-1*_env_*_86–561_CpG	1	36	43
HIV-1*_env_*_611–1014_CpG	4	48	52
HIV-1*_env_*_86–1014_CpG	5	84	95
HIV-1–IRES-GFP	NA	96	NA
HIV-1–IRES-*Renilla*	NA	96	NA
HIV-1Gag-Venus	NA	64	NA
HIV-1Gag-STOP-Venus	NA	64	NA

a NA, not applicable.

We also combined the two regions in *env* containing 36 and 48 CpGs for a total of 84 CpGs (HIV-1*_env_*_86–1014_CpG) (Fig. 3A and Table 1). This inhibited HIV-1 infectious-virus production in an approximately additive manner compared to the two regions’ individual effects through both ZAP-dependent and ZAP-independent effects on genomic-RNA abundance and viral protein expression (Fig. 3C and E). To determine the contribution of decreased Env expression to CpG-mediated inhibition, we pseudotyped the viruses with the vesicular stomatitis virus glycoprotein (VSV-G) and found that the ZAP-dependent and -independent defects in infectious-virus production were still present (Fig. 3D).

**FIG 4:**
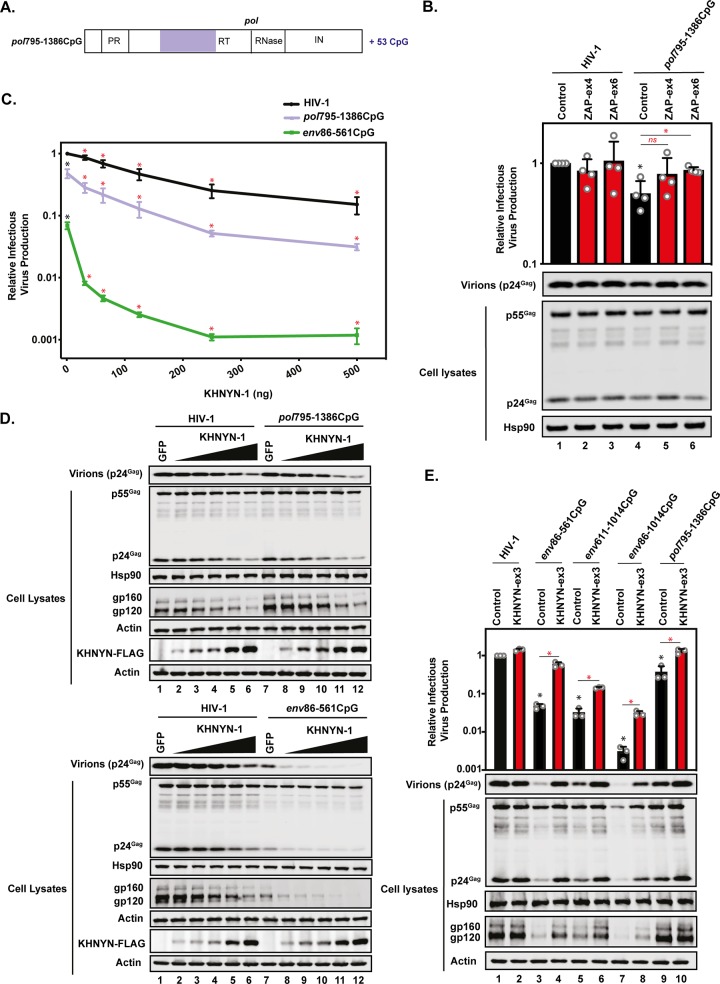
Introduction of 53 CpGs into *pol* only moderately sensitizes the virus for ZAP- and KHNYN-dependent inhibition of infectious-virus production. (A) Schematic representation of HIV-1 *pol* with the region of synonymously introduced CpGs in HIV-1*_pol_*_795–1386_CpG highlighted. (B) HeLa control, ZAP-ex4, and ZAP-ex6 CRISPR cells were transfected with pHIV-1 or pHIV-1*_pol_*_795–1386_CpG plus pGFP. Viral infectivity was measured using TZM-bl reporter cells infected with cell culture supernatants. Gag expression in the media, as well as Gag and Hsp90 expression in the cell lysates, was detected by immunoblotting. The bar charts show the average values of the results of four independent experiments normalized to the values obtained for wild-type HIV-1 in the HeLa control CRISPR cells. The black asterisk compares the virus containing introduced CpGs in the control CRISPR cells to wild-type HIV-1 in the control CRISPR cells. The red asterisk/ns compares the virus containing introduced CpGs between the ZAP CRISPR cells (red bars) and the control CRISPR cells (black bars). (C and D) HeLa cells were transfected with 500 ng pHIV-1, pHIV-1_EnvCpG86–561_, or pHIV-1*_pol_*_795–1386_CpG and 500 ng of pGFP-FLAG or 31.25 ng, 62.5 ng, 125 ng, 250 ng, or 500 ng pKHNYN-1-FLAG plus the amount of pGFP-FLAG required to make 500 ng total. (C) Viral infectivity was measured using TZM-bl reporter cells infected with cell culture supernatants. Each point shows the average value of the results of three independent experiments normalized to the value obtained for wild-type HIV-1 in HeLa cells. *, *P* < 0.05; ns, not significant, as determined by a two-tailed unpaired *t* test. The black asterisks/ns compare the virus containing introduced CpGs to wild-type HIV-1 with 0 ng of KHNYN. The red asterisks/ns compare the virus containing introduced CpGs between the points of KHNYN overexpression to 0 ng of KHNYN. (D) Gag expression in the media, as well as Gag, Hsp90, Env, actin, and KHNYN-FLAG expression in the cell lysates, was detected using immunoblotting. (E) HeLa control and KHNYN-ex3 CRISPR cells were transfected with pHIV-1 or pHIV-1*_env_*_86–561_CpG, pHIV-1*_env_*_611–1014_CpG, pHIV-1*_env_*_86–1014_CpG, or pHIV-1*_pol_*_795–1386_CpG plus pGFP. Viral infectivity was measured using TZM-bl reporter cells infected with cell culture supernatants. Gag expression in the media, as well as Gag, Hsp90, Env. and actin expression in the cell lysates, was detected using immunoblotting. The bar charts show the average values of the results of three independent experiments normalized to the values obtained for wild-type HIV-1 in the HeLa control CRISPR cells. The error bars represent standard deviations. *, *P* < 0.05 as determined by a two-tailed unpaired *t* test. The black asterisks compare the virus containing introduced CpGs in the control CRISPR cells to wild-type HIV-1 in the control CRISPR cells. The red asterisks compare the virus containing introduced CpGs between the KHNYN CRISPR cells (red bars) and the control CRISPR cells (black bars).

We then introduced 53 CpGs into a region of *pol* that did not contain any known or detectable *cis*-acting elements (HIV-1*_pol_*_795–1386_CpG) (Fig. 2 and 4A and Table 1; see Data Set S2). Surprisingly, this large number of CpGs caused only a small (∼2-fold) reduction in Gag expression and infectious-virus production in control CRISPR cells, and this effect was eliminated in the ZAP knockout cells (Fig. 4B). This suggests that the magnitude of ZAP-dependent restriction is not simply proportional to the absolute number of CpGs added to the viral genome.

We have recently identified KHNYN as an essential ZAP cofactor for CpGs to inhibit HIV-1*_env_*_86–561_CpG gene expression and infectious-virus production (31). Our previous work showed that KHNYN overexpression inhibited HIV-1*_env_*_86–561_CpG much more potently than wild-type HIV-1, indicating that the introduced CpGs were required for KHNYN to inhibit HIV-1 (31). To determine if inhibition by KHNYN correlated with CpG abundance or ZAP sensitivity, we overexpressed KHNYN on wild-type HIV-1, HIV-1*_env_*_86–561_CpG, or HIV-1*_pol_*_795–1386_CpG (Fig. 4C and D). KHNYN antiviral activity was correlated with the sensitivity of the virus to endogenous ZAP, with HIV-1*_env_*_86–561_CpG inhibited much more potently than HIV-1*_pol_*_795–1386_CpG or wild-type HIV-1. We also tested whether endogenous KHNYN was required to restrict HIV-1*_env_*_611–1014_CpG, HIV-1*_env_*_86–1014_CpG, and HIV-1*_pol_*_795–1386_CpG using the KHNYN CRISPR cells that we previously characterized (31). Wild-type HIV-1 infectious-virus production was not affected by depleting KHNYN, while HIV-1*_env_*_86–561_CpG infectious-virus production and gene expression were substantially increased (31) (Fig. 4E). The CpG-mediated inhibition of HIV-1*_env_*_611–1014_CpG and HIV-1*_env_*_86–1014_CpG infectious-virus production was partially rescued in KHNYN CRISPR cells, which was correlated with their restriction by ZAP (Fig. 3C and 4E). The small decrease in infectious-virus production for HIV-1*_pol_*_795–1386_CpG was rescued in the KHNYN CRISPR cells, indicating that the 53 introduced CpGs in *pol* moderately inhibited HIV-1 through ZAP and KHNYN (Fig. 4E).

**FIG 5:**
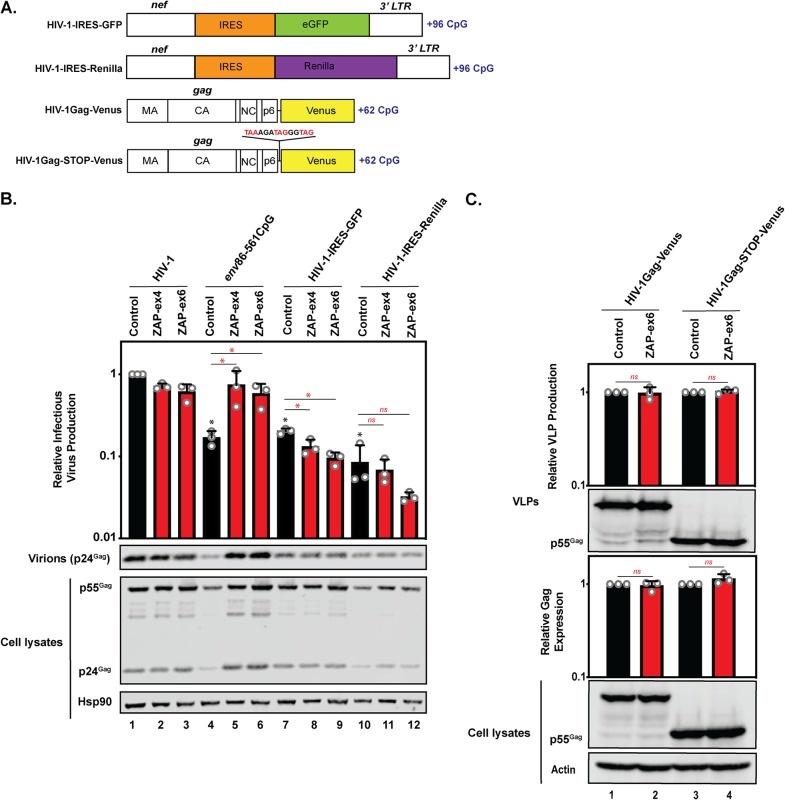
HIV-1–IRES-GFP, HIV-1–IRES-*Renilla*, and HIV-1 containing a Gag-Venus fusion protein are not targeted by endogenous levels of ZAP in HeLa cells. (A) Schematic representation of the HIV-1 *nef*/long terminal repeat (LTR) region with the introduced GFP and *Renilla* reporter genes and HIV-1 Gag-Venus fusion constructs. The nucleotide sequence shows the insertion between the *gag* and *venus* open reading frames that introduces stop codons (highlighted in red). (B) Control, ZAP-ex4, and ZAP-ex6 CRISPR cells were transfected with pHIV-1, HIV-1*_env_*_86–561_CpG, pHIV-1-IRES-GFP, or pHIV-1-IRES-*Renilla* plus pGFP. Infectious-virus production was measured using TZM-bl reporter cells infected with cell culture supernatants. Gag expression in the media, as well as Gag and Hsp90 expression in the cell lysates, was detected using immunoblotting. The bar chart shows the average values of the results of three independent experiments normalized to the values obtained for wild-type HIV-1 in HeLa control CRISPR cells. (C) Control and ZAP-ex6 CRISPR cells were transfected with pHIV-1Gag-Venus or pHIV-1Gag-STOP-Venus. Gag expression in the media, as well as Gag and Hsp90 expression in the cell lysates, was detected using immunoblotting. The bar chart shows the average values of the results of three independent experiments normalized to the virus in control CRISPR cells. The error bars represent standard deviations. *, *P* < 0.05; ns, not significant, as determined by a two-tailed unpaired *t* test. The black asterisks/ns compare the virus containing introduced CpGs in the control CRISPR cells to wild-type HIV-1 in the control CRISPR cells. The red asterisks/ns compare the virus containing introduced CpGs between the ZAP CRISPR cells (red bars) and the control CRISPR cells (black bars).

HIV-1 strains containing reporter genes, such as those encoding green fluorescent protein (GFP) or luciferase, are important experimental tools and have large numbers of CpGs in the reporter gene. Therefore, it was important to determine whether ZAP inhibits these viruses, because this could confound the interpretation of results obtained under some experimental conditions. Specifically, we analyzed whether ZAP could restrict HIV-1 containing the encephalomyocarditis virus (EMCV) internal ribosome entry site (IRES) followed by enhanced GFP (eGFP) (IRES-GFP) or *Renilla* luciferase (IRES-*Renilla*) (Fig. 5A and Table 1) (41, 42). Both IRES-GFP and IRES-*Renilla* introduced 96 CpGs into the viral genome. While both reporter viruses produced less Gag and infectious virus than wild-type HIV-1, ZAP depletion did not increase this production (Fig. 5B). We also analyzed the effect of a Venus fluorescent protein-plus-inker sequence that introduced 64 CpGs as a fusion protein with Gag (Fig. 5A and Table 1) (43). Interestingly, this sequence did not sensitize Gag abundance or virus-like particle (VLP) production to ZAP (Fig. 5C). Because a ribosome could displace ZAP bound to CpGs in an open reading frame as it moves along the mRNA, it is possible that CpGs in a 3′ untranslated region (UTR) could inhibit HIV-1 gene expression in a ZAP-dependent manner more effectively than CpGs in a coding region. Therefore, we inserted stop codons between Gag and Venus in both the Gag and Pol reading frames (Fig. 5A and Table 1). However, the CpGs in the context of the 3′ UTR also did not promote ZAP-mediated inhibition of Gag expression or VLP production (Fig. 5C). In sum, the total number of CpGs introduced into the HIV-1 genome does not correlate with their antiviral activity in the context of endogenous ZAP levels in HeLa cells.

### Increasing ZAP abundance inhibits CpG-containing HIV-1.

**FIG 6:**
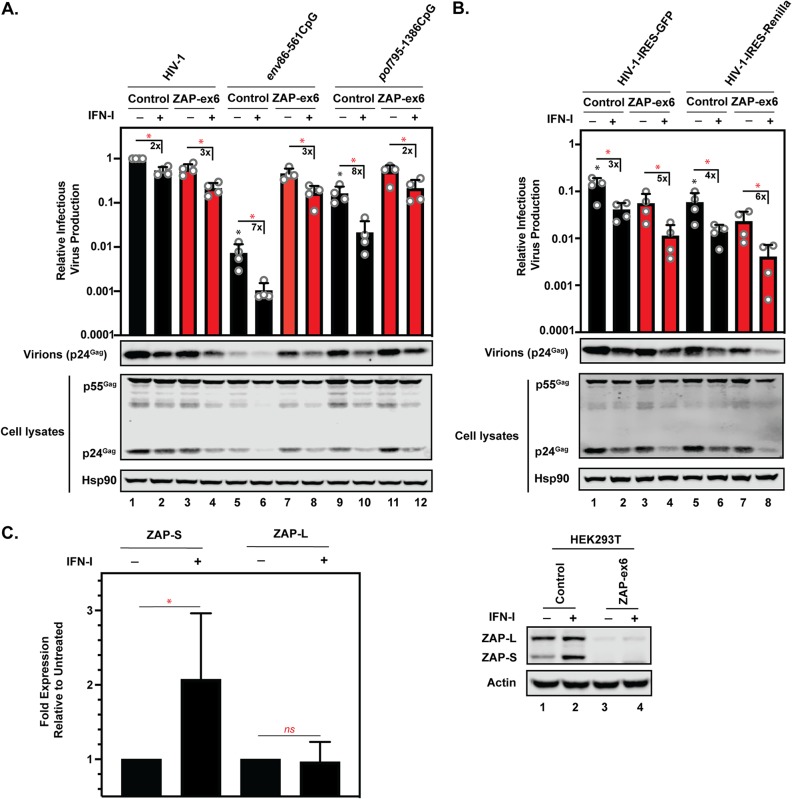
Type I interferon inhibits HIV-1 containing introduced CpG dinucleotides using ZAP-dependent and ZAP-independent mechanisms and induces an ∼2-fold increase in ZAP-S expression in 293T cells. (A and B) HEK293T control and ZAP-ex6 CRISPR cells were transfected with pHIV-1, pHIV-1*_env_*_86–561_CpG, pHIV-1*_pol_*_795–1386_CpG, pHIV-1-IRES-GFP, or pHIV-1-IRES-*Renilla* plus pGFP with (+) or without (−) 1,000 U/ml of type I interferon treatment. Infectious-virus production was measured using TZM-bl reporter cells infected with cell culture supernatants. Gag expression in the media, as well as Gag and Hsp90 expression in the cell lysates, was detected using immunoblotting. The bar charts show the average values of the results of four independent experiments normalized to wild-type HIV-1 in HEK293T control CRISPR cells. The numbers above the bars represent the fold decrease in relative infectious-virus production due to the type I interferon treatment. The error bars represent standard deviations. *, *P* < 0.05; ns, not significant, as determined by a two-tailed unpaired *t* test. The black asterisks/ns compare the virus containing introduced CpGs in the control CRISPR cells to wild-type HIV-1 in the control CRISPR cells. The red asterisks/ns compare results without and with IFN-I treatment. The black bars are samples in control CRISPR cells and the red bars are samples in ZAP CRISPR cells. (C) Bar chart showing the average values of the results of 15 independent 1,000-U/ml interferon treatments for ZAP-S and ZAP-L expression with a representative Western blot for ZAP expression in HEK293T control and ZAP-ex6 CRISPR cells with and without type I interferon treatment. *, *P* < 0.05; ns, not significant, as determined by a two-tailed unpaired *t* test.

We then analyzed whether increasing ZAP abundance further inhibited HIV-1 containing introduced CpGs. First, we treated control and ZAP knockout HEK293T cells with type I interferon (IFN-I) (Fig. 6). Similar to previous reports, this consistently increased expression of the short isoform of ZAP (ZAP-S) by ∼2-fold and had no substantial effect on expression of the long isoform of ZAP (ZAP-L) (Fig. 6C) (44, 45). IFN-I inhibited all of the viruses. For wild-type HIV-1, the magnitudes of inhibition were similar in the control and ZAP knockout cells (Fig. 6A), which is consistent with the observation that endogenous ZAP does not target wild-type HIV-1 (16, 31). The magnitude of IFN-I inhibition of HIV-1*_env_*_86–561_CpG was reduced when ZAP was depleted, indicating that ZAP contributes to the antiviral effect of IFN-I on the virus. Importantly, IFN-I treatment augmented the ZAP-dependent inhibition of HIV-1*_pol_*_795–1386_CpG. This suggests that CpG-rich sequences in an HIV open reading frame that are only weakly restricted by endogenous ZAP levels can be further sensitized by IFN-I. However, even in the presence of IFN-I, the magnitude of inhibition by the 53 introduced CpGs in *pol* was lower than that of the inhibition mediated by the 36 introduced CpGs in *env*. For HIV-1–IRES-GFP and HIV-1–IRES-*Renilla*, the magnitude of IFN-I inhibition was not decreased upon ZAP depletion (Fig. 6B). This indicates that endogenous ZAP does not target these viruses, despite the large numbers of CpGs that have been introduced into the viral genome.

**FIG 7:**
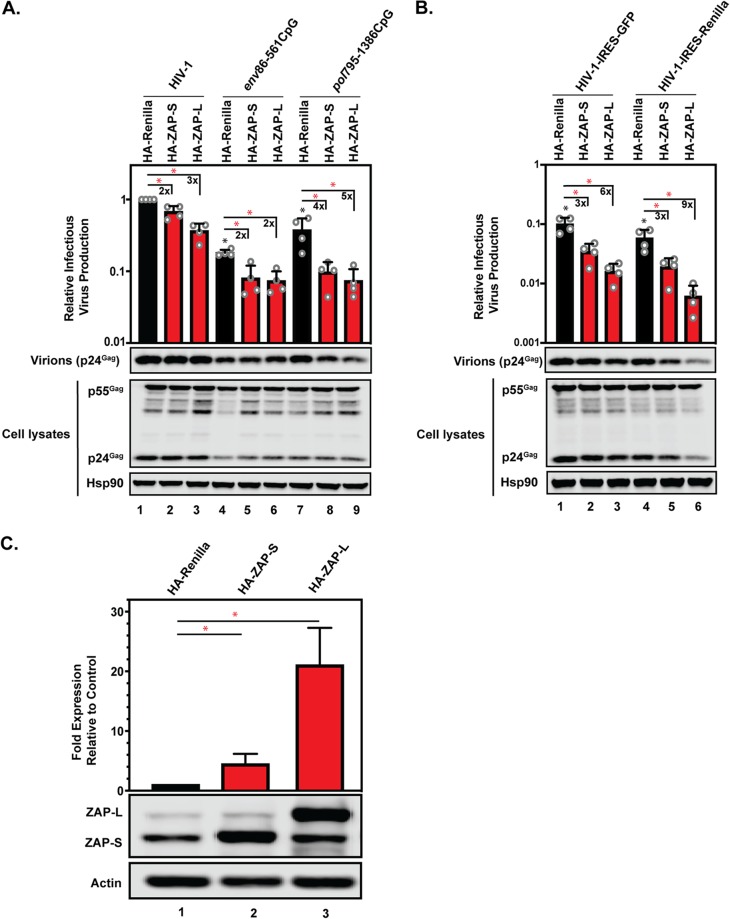
ZAP overexpression inhibits wild-type HIV-1 and HIV-1 containing introduced CpG dinucleotides. (A and B) HeLa cells were transfected with pHIV-1, pHIV-1*_env_*_86–561_CpG, pHIV-1*_pol_*_795–1386_CpG, pHIV-1-IRES-GFP, or pHIV-1-IRES-*Renilla* plus either pHA-*Renilla*, pHA-ZAP-S, or pHA-ZAP-L. Infectious-virus production was measured using TZM-bl reporter cells infected with cell culture supernatants. Gag expression in the media, as well as Gag and Hsp90 expression in the cell lysates, was detected using immunoblotting. The bar charts show the average values of the results of four independent experiments normalized to wild-type HIV-1 plus HA-*Renilla*. The numbers above the bars represent the fold decrease in relative infectious-virus production due to ZAP-S or ZAP-L overexpression. (C) Bar chart and representative Western blot for ZAP expression showing the average ZAP abundance for 15 independent transfections of p-HA-ZAP-S or pHA-ZAP-L in HeLa cells. The error bars represent standard deviations. *, *P* < 0.05 as determined by a two-tailed unpaired *t* test. The black asterisks compare the virus containing introduced CpGs in the control CRISPR cells to wild-type HIV-1 in the control CRISPR cells. The red asterisks compare the virus with HA-*Renilla* overexpression (black bars) to HA-ZAP overexpression (red bars).

Because IFN-I only moderately upregulated ZAP-S, we also overexpressed ZAP-S or ZAP-L in HeLa cells. This increased ZAP-S expression ∼5-fold and ZAP-L expression ∼20-fold (Fig. 7C). Both ZAP isoforms inhibited all of the viruses tested at least 2-fold (Fig. 7). Interestingly, at high levels of ZAP-S or ZAP-L, the 36 introduced CpGs in *env* and the 53 CpGs in *pol* inhibited HIV-1 infectious-virus production to similar levels (Fig. 7A). Both HIV-1–IRES-GFP and HIV-1–IRES-*Renilla* were potently inhibited by overexpressed ZAP, indicating that when ZAP abundance is high enough, the CpGs introduced into these viruses can be targeted (Fig. 7B). Therefore, the inhibition observed for HIV-1–IRES-GFP and HIV-1–IRES-*Renilla* at high ZAP levels indicates that CpGs in contexts that are not targeted by the endogenous ZAP levels in HeLa cells (Fig. 4) can be targeted if ZAP abundance is substantially increased (Fig. 7B).

### CpG dinucleotides introduced into the 5′ end of *gag* inhibit HIV-1 replication in a ZAP-independent manner.

**FIG 8:**
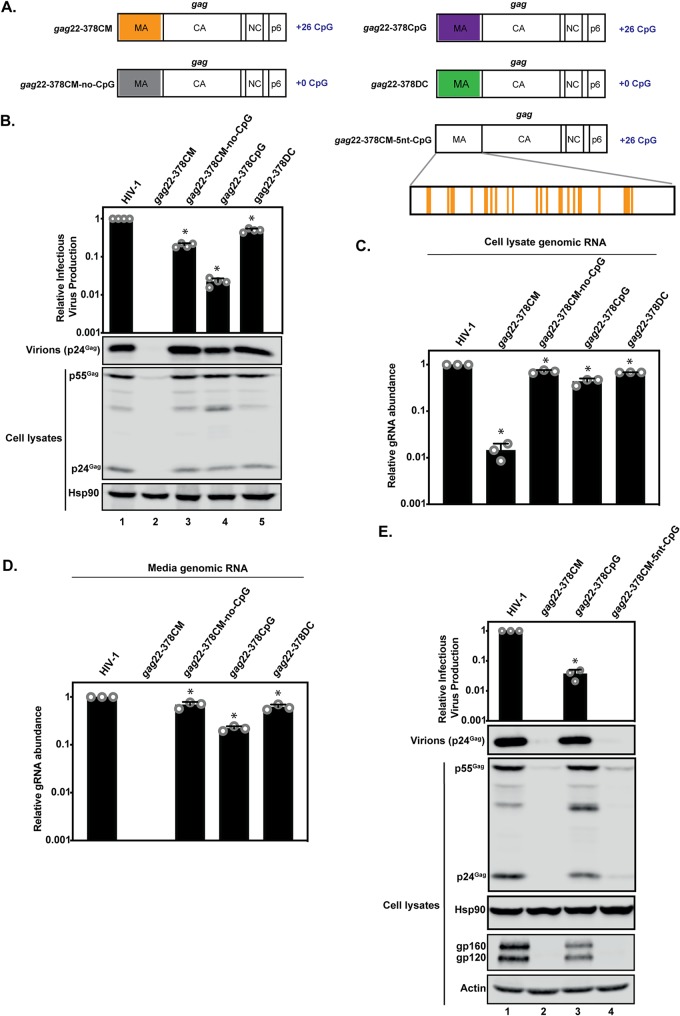
Introduction of CpG dinucleotides into *gag* nt 22 to 378 inhibits HIV-1 infectious-virus production. (A) Schematic representation of HIV-1 *gag* with introduced synonymous mutations in HIV-1*_gag_*_22–378_CM, HIV-1*_gag_*_22–378_CpG, HIV-1*_gag_*_22–378_CM–no-CpG, HIV-1*_gag_*_22–378_DC, and HIV-1*_gag_*_22–378_CM–5nt-CpG highlighted. (B to E) HeLa cells were transfected with pHIV-1, pHIV-1*_gag_*_22–378_CM, pHIV-1*_gag_*_22–378_CM–no-CpG, pHIV-1*_gag_*_22–378_CpG, or pHIV-1*_gag_*_22–378_DC (B to D) or pHIV-1, pHIV-1*_gag_*_22–378_CM, pHIV-1*_gag_*_22–378_CpG, or pHIV-1*_gag_*_22–378_CM–5nt-CpG and pGFP (E). The culture supernatants were used to infect TZM-bl reporter cells to measure the amount of infectious-virus production. (B and E) Gag expression in the media, as well as Gag, Hsp90, Env, and actin expression in the cell lysates, was detected using immunoblotting. (C and D) Genomic-RNA abundance was quantified by qRT-PCR in cell lysates (C) and media (D). The bar charts show the average values of the results of three (B and E) or four (C and D) independent experiments normalized to the value obtained for wild-type HIV-1. The error bars represent standard deviations. *, *P* < 0.05 as determined by a two-tailed unpaired *t* test comparing the virus containing synonymous mutations to wild-type HIV-1.

We previously introduced CpG dinucleotides into nt 22 to 378 of *gag* in two different contexts (Fig. 8A and Table 1) and found that they inhibited viral replication with different phenotypes on Gag expression (15). For HIV-1*_gag_*_22–378_CM, the codon modified (CM) sequence was derived from a codon-optimized Gag-Pol construct and introduced 109 synonymous nucleotide changes and 26 CpGs (Table 1) (15, 46). In the context of a single-cycle infectivity assay, HIV-1*_gag_*_22–378_CM Gag expression and infectious-virus production were decreased to the limit of detection (Fig. 8B). This correlated with a large decrease in genomic-RNA abundance in the cell lysate and medium (Fig. 8C and D). The CpG dinucleotides were necessary to inhibit the virus, because when they were removed (while leaving the 79 mutations that did not introduce a CpG dinucleotide) to create HIV-1*_gag_*_22–378_CM–no-CpG (Fig. 8A and Table 1), gRNA abundance, Gag expression, and infectious-virion production were substantially increased (Fig. 8B). However, when the 26 CpG dinucleotides were introduced without the additional mutations in the codon-optimized Gag sequence to create HIV-1*_gag_*_22–378_CpG (Fig. 8A and Table 1), infectious-virus production was decreased by >95%, even though there was no substantial decrease in Gag expression (Fig. 8B).

Deletion of nt 22 to 378 in *gag* does not substantially decrease infectious-virus production (15, 47), indicating that there are no essential *cis*-acting elements in the region. However, altering the RNA sequence could modulate the local RNA structure in ways that a large deletion did not, and the 5′ region of *gag* has been shown to indirectly regulate gRNA packaging by regulating the structure of the 5′ UTR (48, 49). To determine if the CpGs in HIV-1*_gag_*_22–378_CpG specifically decreased infectious-virus production or if a *cis*-acting regulatory element in the region had been mutated, we changed the codons previously mutated to introduce CpGs into a different codon (DC) that was not the wild-type HIV-1 codon and did not introduce a CpG where possible to produce pHIV-1*_gag_*_22–378_DC (Fig. 8A and Table 1). HIV-1*_gag_*_22–378_DC produced levels of infectious virus similar to those of wild-type HIV-1 (Fig. 8B). We also measured gRNA abundance in the cell lysate and medium. HIV-1*_gag_*_22–378_CpG had ∼60% and ∼80% decreases in gRNA in the lysate and medium, respectively, while HIV-1*_gag_*_22–378_DC had levels of gRNA similar to those of wild-type HIV-1 (Fig. 8C and D). This indicates that the introduced CpGs are necessary for the reduction in infectious-virus production and not a result of mutating essential *cis*-acting elements in the region that modulate gRNA packaging or other steps of HIV-1 replication. In sum, while HIV-1*_gag_*_22–378_CM and HIV-1*_gag_*_22–378_CpG have the same 26 introduced CpGs, there is a much larger decrease in Gag expression and intracellular gRNA abundance for HIV-1*_gag_*_22–378_CM, and this is due to the introduced CpGs. This suggests that the sequence surrounding the CpG dinucleotides modulates their inhibitory effect.

To test the effect of the sequence proximal to the CpG, we changed all of the mutations in HIV-1*_gag_*_22–378_CM that were not within 5 nucleotides of an introduced CpG back to the wild-type HIV-1 sequence to produce HIV-1*_gag_*_22–378_CM–5nt-CpG (Fig. 8A and Table 1). Interestingly, HIV-1*_gag_*_22–378_CM–5nt-CpG Gag expression and infectious-virus production were very similar to those of HIV-1*_gag_*_22–378_CM (Fig. 8E). This indicates that the sequence immediately surrounding the CpG dinucleotides influences the degree to which they inhibit HIV-1 gene expression. We also analyzed intracellular Env abundance and found that Env expression was reduced to undetectable levels for HIV-1*_gag_*_22–378_CM and HIV-1*_gag_*_22–378_CM–5nt-CpG (Fig. 8E). Env expression was also decreased for HIV-1*_gag_*_22–378_CpG, which likely accounts for the decreased infectious-virus production, in addition to the reduced gRNA levels present in virions (Fig. 8D). The decrease in Env expression was unexpected, because the region in the HIV-1 genome containing the introduced CpGs in these viruses is present only in the unspliced transcript encoding Gag and Gag-Pol and not in the singly spliced *env* mRNAs. Overall, the local sequence context of the CpGs in *gag* nt 22 to 378 determines the magnitude of inhibition for both Gag and Env expression.

**FIG 9:**
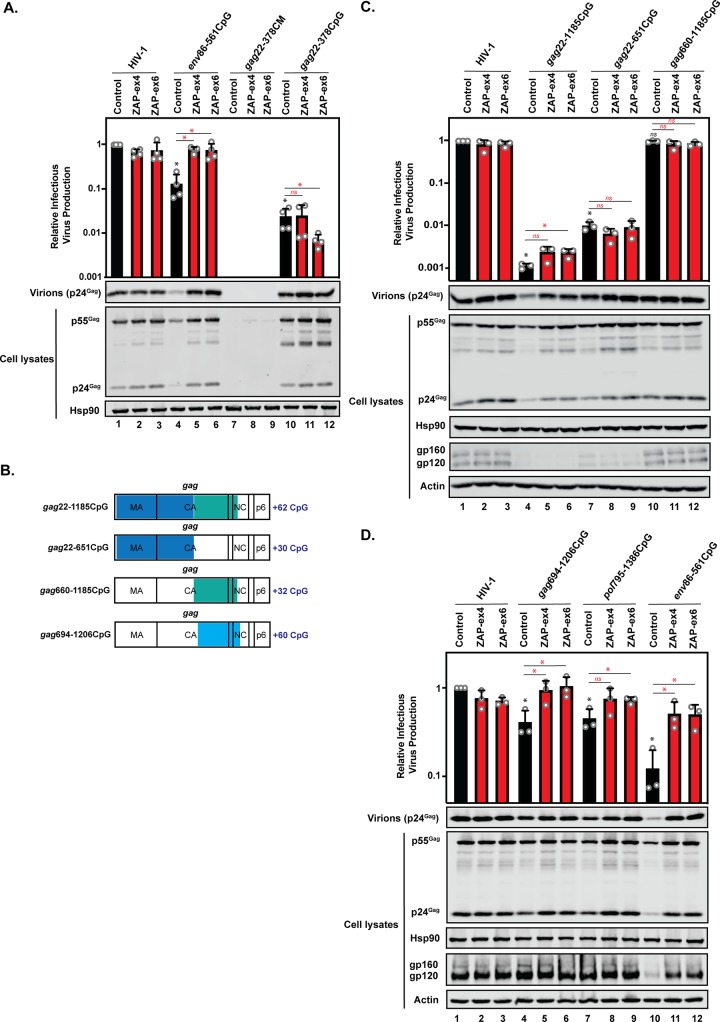
CpG dinucleotides in *gag* have ZAP-dependent and ZAP-independent effects, but the number of CpGs is not correlated with antiviral activity. (A, C, and D) HeLa control, ZAP-ex4, and ZAP-ex6 CRISPR cells were transfected with pHIV-1, pHIV-1*_env_*_88-561_CpG, pHIV-1*_gag_*_22–378_CM, or pHIV-1*_gag_*_22–378_CpG (A); pHIV-1, pHIV-1_g_*_ag_*_22–1185_CpG, pHIV-1*_gag_*_22–651_CpG, or pHIV-1*_gag660_*_-1185_CpG (C); or pHIV-1, pHIV-1*_env_*_88-561_CpG, pHIV-1*_pol_*_795–1386_CpG, or pHIV-1*_gag_*_694–1206_CpG plus pGFP (D). The culture supernatants were used to infect TZM-bl reporter cells to measure infectious-virus production. Gag expression in the media, as well as Gag, Hsp90, Env, and actin expression in the cell lysates, was detected using immunoblotting. The bar charts show the average values of the results of three (C and D) or four (A) independent experiments normalized to HIV-1 in HeLa control CRISPR cells. The error bars represent standard deviations. *, *P* < 0.05; ns, not significant, as determined by a two-tailed unpaired *t* test. The black asterisks/ns compare the virus containing introduced CpGs in the control CRISPR cells to wild-type HIV-1 in the control CRISPR cells. The red asterisks/ns compare the virus containing introduced CpGs between the ZAP CRISPR cells (red bars) and the control CRISPR cells (black bars). (B) Schematic representation of HIV-1 *gag* with the regions of synonymously introduced CpGs in HIV-1_g_*_ag_*_22–1185_CpG, HIV-1*_gag_*_22–651_CpG, HIV-1*_gag_*_660-1185_CpG, and HIV-1*_gag_*_694–1206_CpG highlighted.

We then analyzed whether ZAP was necessary for the CpGs in HIV-1*_gag_*_22–378_CM and HIV-1*_gag_*_22–378_CpG to inhibit Gag expression and infectious-virus production. In contrast to HIV-1*_env_*_86–561_CpG, ZAP depletion did not increase infectious-virus production for HIV-1*_gag_*_22–378_CM and HIV-1*_gag_*_22–378_CpG (Fig. 9A). This shows that the CpGs introduced into the 5′ region of *gag* inhibit HIV-1 gene expression and infectious-virus production through a ZAP-independent mechanism. To determine if introducing a larger number of CpGs into *gag* could make it ZAP sensitive, we cloned pHIV-1*_gag_*_22–1188_CpG, which contains 62 CpG dinucleotides distributed across ∼1,100 nt (Fig. 9B and Table 1). In addition, we produced pHIV-1*_gag_*_22–654_CpG and HIV-1*_gag_*_658–1188_CpG, which contain 30 and 32 CpGs, respectively. Introducing CpGs into nt 22 to 654 of *gag* led to large decreases in Gag expression, Env expression, and infectious-virus production (Fig. 9C). Interestingly, Gag expression for HIV-1*_gag_*_22–1188_CpG and HIV-1*_gag_*_22–654_CpG was increased in the ZAP knockout cells, though this did not affect Env expression or infectious-virus production. In contrast, introduction of 32 CpGs into nt 658 to 1188 of *gag* had no effect on Gag expression, Env expression, or infectious-virus production (Fig. 9C). Therefore, we introduced 60 CpGs into this 3′ region of gag (HIV-1*_gag_*_694–1206_CpG) (Fig. 9B and Table 1) and analyzed the effect on infectious-virus production in control and ZAP CRISPR cells (Fig. 9D). The 60 CpGs in the 3′ region of *gag* inhibited HIV-1 infectious-virus production about 2-fold, which is similar to the effect that 53 CpGs had in *pol* and less than the 36 CpGs in *env*. Overall, in some contexts, CpGs introduced into *gag* allow ZAP to inhibit Gag expression, but the number of CpGs is not correlated with the magnitude of the inhibitory effect. Furthermore, the CpGs introduced into the 5′ region of *gag* inhibit infectious-virus production in a ZAP-independent manner.

### CpG dinucleotides introduced into *gag* can inhibit HIV-1 gene expression by modulating pre-mRNA splicing.

**FIG 10:**
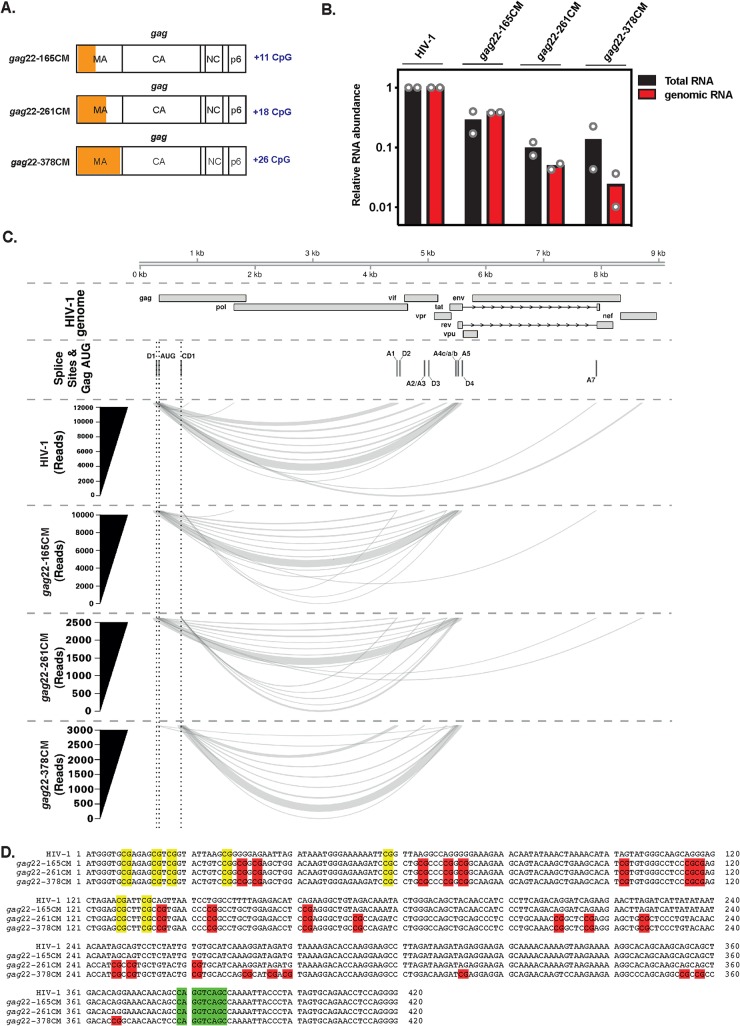
Codon modification in the 5′ end of *gag* activates a cryptic splice donor. (A) Schematic representation of the HIV-1 *gag* region with introduced synonymous mutations in HIV-1*_gag_*_22–165_CM, HIV-1*_gag_*_22–261_CM, or HIV-1*_gag_*_22–378_CM highlighted. (B) HeLa cells were transfected with pHIV-1, pHIV-1*_gag_*_22–165_CM, pHIV-1*_gag_*_22–261_CM, or pHIV-1*_gag_*_22–378_CM. Total and genomic-RNA abundances were quantified by qRT-PCR in the cell lysates. The bar charts show the average values of the results of two independent experiments normalized to wild-type HIV-1. (C) Relative usage of splice donor 1 and cryptic splice donor 1 upon codon modification of HIV-1. The 9,173-nt HIV-1 genomic RNA and features are depicted in the “HIV-1 genome” track. Canonical donors (D1 to D4) and acceptors (A1 to A7), a codon modification-induced cryptic donor (CD1), and the *gag* start codon (AUG) are shown in the “Splice Sites and Gag AUG” track. The number of reads supporting use of D1 (nt 291) or CD1 (nt 718) paired with a given splice acceptor is depicted by line width (*y* axis). The splice data line height is arbitrary. The read numbers shown are the sums across duplicate samples per thousand HIV-mapping reads. (D) Alignment of *gag* sequences for HIV-1, HIV-1*_gag_*_22–165_CM, HIV-1*_gag_*_22–261_CM, and HIV-1*_gag_*_22–378_CM. Introduced CpGs are highlighted in red, and CpGs found in the wild-type HIV-1 *gag* sequence are highlighted in yellow. The cryptic splice donor (CD1) activated by the synonymous mutations in *gag* nt 22 to 378 is highlighted in green.

The HIV-1 genomic RNA undergoes extensive alternative splicing to mediate expression of all of the viral genes, and synonymous mutations in *gag* have previously been shown to disrupt HIV-1 splicing (36, 50). Since the CpGs in the 5′ region of *gag* reduced Env expression in all sequence contexts tested (Fig. 8E and 9C), we speculated that they could affect splicing. Therefore, we analyzed RNA abundance when progressively longer regions of codon-optimized *gag* sequence were added to the virus, which introduced 11, 18, or 26 CpGs (HIV-1*_gag_*_22–165_CM, HIV-1*_gag_*_22–261_CM, and HIV-1*_gag_*_22–378_CM) (Fig. 10A and Table 1). We have previously analyzed these viruses and have shown that they are deficient for genomic-RNA abundance and Gag expression (15). A comparison of the total RNA and the genomic RNA indicated that the abundance of both was decreased by the synonymous mutations (Fig. 10B). To determine whether the decrease in viral RNA abundance is due to altered splicing, we used RNA-seq to sequence the transcriptome in each sample and determine which splice sites were used to produce the mRNAs for each virus (Fig. 10C and Table 2; see Data Set S3 in the supplemental material). This analysis showed that a preexisting cryptic splice donor (CD1) was activated. Importantly, this donor is outside the region into which the CpGs were introduced (Fig. 10D). The frequency of use of the cryptic splice donor increased with the length of the codon-optimized *gag* sequence and coincided with a decrease in the utilization of canonical splice donor 1 (SD1). Activation of the cryptic splice donor increased the length of the first exon incorporated into all of the spliced viral RNAs to include the *gag* sequence prior to CD1. This led to the incorporation of the Gag initiation codon in every transcript upstream of the canonical start codon for the encoded protein (Fig. 10C and D), which is predicted to result in inefficient translation of all HIV-1 proteins encoded by a singly or fully spliced mRNA, including Tat and Rev. This could account for the decrease in total RNA levels, genomic-RNA levels, and Gag and Env expression that we observed for HIV-1*_gag_*_22–378_CM (Fig. 8 and 10) (15).

**TABLE 2:** Splicing events from splice donor 1 or cryptic splice donor 1 to the canonical HIV-1 splice acceptors as a percentage of total HIV-1 splicing events

Start (nt)	End (nt)	Splice sites^*a*^	% of total events for HIV-1							
			WT.1	WT.2	CM22–165.1	CM22–165.2	CM22–261.1	CM22–261.2	CM22–378.1	CM22–378.2
291	4460	D1-A1	11.9	11.9	3.0	3.4	2.5	1.5	0.2	0.0
291	4937	D1-A2	4.4	4.4	3.2	3.3	2.9	2.1	0.0	0.0
291	5324	D1-A3	3.0	3.1	3.4	3.5	4.0	3.1	0.0	0.0
291	5483	D1-A4c	1.2	1.4	0.6	1.1	0.1	0.4	0.0	0.0
291	5501	D1-A4a	4.1	3.7	1.9	2.0	1.6	0.9	0.0	0.0
291	5507	D1-A4b	2.2	2.2	1.7	1.6	1.1	1.1	0.3	0.1
291	5523	D1-A5	25.7	26.2	30.4	33.3	29.7	26.2	0.7	0.9
291	7916	D1-A7	0.9	1.0	0.9	0.8	0.0	0.4	0.0	0.0
718	4460	CD1-A1	0.0	0.0	0.6	0.4	2.0	1.5	7.8	7.4
718	4937	CD1-A2	0.0	0.0	0.0	0.0	0.5	0.8	1.7	1.4
718	5324	CD1-A3	0.0	0.0	0.1	0.1	1.4	1.1	1.6	1.8
718	5483	D1-A4c	0.0	0.0	0.0	0.0	0.0	0.0	0.0	0.0
718	5501	CD1-A4a	0.0	0.0	0.0	0.0	0.0	0.1	1.0	1.2
718	5507	CD1-A4b	0.0	0.0	0.0	0.0	0.4	0.4	3.2	3.0
718	5523	CD1-A5	0.0	0.0	0.2	0.4	4.7	4.4	24.9	23.1
718	7916	CD1-A7	0.0	0.0	0.0	0.0	0.0	0.0	0.0	0.0

a D1, splice donor 1; CD1, cryptic splice donor 1.

## DISCUSSION

There is selection against CpG dinucleotides in many vertebrate RNA viruses, and introducing them into viral genomes may allow novel vaccines to be developed (1–5, 9, 10). However, to attenuate viral replication, it is unclear how many CpGs are required, whether there is an optimal location to insert CpGs, and whether all CpGs inhibit via ZAP. Due to the profound suppression of CpG abundance in HIV-1, we used it as a model system to analyze how CpG dinucleotides inhibit viral replication.

Our results show that CpGs can inhibit HIV-1 replication through at least two independent, but not mutually exclusive, mechanisms. First, they can recruit ZAP and target the viral RNA for degradation. Second, they can inhibit replication by altering pre-mRNA splicing. In addition, CpGs could silence HIV-1 transcription through DNA methylation. The multiple mechanisms by which CpGs inhibit HIV-1 infectious-virus production may explain why they are strongly suppressed in the virus, even compared to other RNA viruses, and may also explain why small changes in the number of CpGs in *env* are linked to disease progression (18). Introducing CpGs into *env* nt 86 to 561 potently inhibits genomic-RNA abundance, Env expression, Gag expression, and infectious-virus production in a ZAP-dependent manner. However, ZAP depletion does not fully rescue infectious-virus production when CpGs are introduced into several other regions of the HIV-1 genome, highlighting the ZAP-independent effects of CpGs, as well as the sensitivity of the 5′ region of *env* for CpGs that mediate ZAP antiviral activity.

We have characterized the ZAP-independent effect for CpGs introduced into the 5′ end of *gag*. These CpGs have a dramatic effect on genomic-RNA levels, Gag expression, and Env expression by promoting the use of a cryptic 5′ splice donor at the expense of SD1. Interestingly, the magnitude of this effect is modulated by the sequence identity immediately surrounding the CpGs. It should be noted that these experiments were done in the context of transiently transfected proviral constructs in HeLa cells, but previous studies have shown that the splicing patterns for HIV-1 are similar in transfected cells and infected T cells (51, 52). The introduced CpGs do not directly enhance the strength of this splice site because they are upstream of the splice donor and do not affect the sequence itself. A previous report has identified that introducing synonymous mutations into the 5′ end of *gag* promotes splicing at this cryptic donor, but the role of CpGs in this was not characterized (50). We found that CpGs introduced into this region have multiple effects on viral replication, including decreases in genomic-RNA stability, Gag expression, virion production, and infectivity per genome (15). All of these phenotypes are likely due to the decreased use of SD1 and the corresponding increase in splicing from the cryptic splice donor in *gag*. The CpGs introduced into the 5′ end of *gag* could inhibit a preexisting exonic splicing silencer (ESS) or introduce an exonic splicing enhancer (ESE). We favor the hypothesis that the CpGs introduced an ESE, because the synonymous mutations promote splicing at the cryptic splice donor in a length-dependent manner and the sequence within 5 nucleotides surrounding the CpG modulates the magnitude of the decrease in Env and Gag expression. However, further experiments will be required in order to characterize how CpGs modulate splicing. There are approximately 1,500 RNA binding proteins in the human genome, most of which do not have a well-characterized recognition sequence, though several have been reported to bind sequences that contain CpGs (53, 54). Therefore, an unknown number of RNA binding proteins bind CpGs, and we do not yet know which protein regulates HIV-1 splicing in a CpG-dependent manner. In addition, introducing CpGs into the HIV-1 genome may affect its local or long-range RNA structures; posttranscriptional modifications, such as cytosine methylation; or other aspects of RNA biology (55–57).

Surprisingly, the magnitude of ZAP-mediated inhibition was not correlated with the number of CpGs introduced into the viral genome, and some regions of the genome can tolerate substantial numbers of CpGs. Thirty-six CpGs inserted into the 5′ region of *env* had the greatest ZAP-dependent inhibitory phenotype. This corresponds to the observations by Takata et al., who first identified this region in a panel of viruses with large numbers of synonymous mutations in different regions of the HIV-1 genome (16, 50). The introduction of 53 CpGs into *pol* or 60 CpGs into the 3′ end of *gag* produced only a small amount of ZAP-dependent inhibition of infectious-virus production. KHNYN overexpression also produced less inhibition of infectious-virus production when 53 CpGs were introduced into *pol* than when 36 CpGs were added to *env*. This supports our hypothesis that KHNYN antiviral activity is controlled by ZAP’s ability to target the viral RNA (31). Only when ZAP levels were very high due to overexpression from a cDNA plasmid did the 53 CpGs in *pol* mediate a level of repression similar to that mediated by the 36 CpGs introduced into *env* nt 86 to 561. This highlights the facts that this region in *env* is very sensitive to the endogenous levels of ZAP in HeLa cells and that the position or local context of the CpG is important for ZAP to inhibit the virus. Interestingly, the weak inhibition of infectious-virus production by the 53 CpGs in *pol* mediated by endogenous ZAP levels could be substantially enhanced by IFN-I treatment. Therefore, part of the anti-HIV activity mediated by IFN-I (58) may be to promote ZAP targeting CpGs in contexts where it normally does so inefficiently. The fact that there is only a small increase in ZAP-S abundance upon IFN-I treatment raises the question of whether this induction of ZAP is sufficient to explain the phenotype or whether increased abundance or activity of ZAP cofactors, such as TRIM25 or KHNYN, may contribute (29–31, 59).

Because HiV-1 strains containing reporter genes are commonly used research tools, we investigated whether the large numbers of CpGs in the EMCV IRES, GFP, or *Renilla* luciferase could sensitize these viruses to ZAP. Adding 96 CpGs to the 3′ end of the genome in the context of IRES-GFP or IRES-*Renilla* luciferase did not sensitize the virus to the endogenous levels of ZAP in HeLa or HEK293T cells. Similarly, HIV-1 with GFP in place of *nef* has previously been shown not to be targeted by endogenous levels of ZAP in HeLa or MT4 cells (16). While HIV-1–IRES-GFP and HIV-1–IRES-*Renilla* were not inhibited by ZAP after IFN-I treatment, they were inhibited when high levels of ZAP were present due to overexpression from a cDNA plasmid. Therefore, ZAP abundance can determine whether CpG-containing viral genomes are targeted, though it is unclear whether the ZAP levels produced from plasmid-based overexpression can be achieved in a relevant *in vivo* context. This suggests that these reporter viruses are useful tools that may not be affected by ZAP under many experimental conditions.

An important area of future research is to determine why CpGs in some contexts or regions are efficiently targeted by ZAP and others are not. To date, the primary evidence that ZAP directly binds CpGs comes from PAR-CLIP experiments (16). The advantage of this technique is that it captures ZAP binding to CpGs in a living cell. However, other cellular factors present could modulate ZAP’s binding specificity. Several groups have shown that the ZAP cofactor TRIM25 can bind cellular and viral RNA, and it has been reported to regulate ZAP binding to Sindbis virus RNA (29, 30, 60–65). Therefore, TRIM25 or other ZAP cofactors could bind specific motifs in viral RNA to determine the sensitivity of the RNA to ZAP-mediated antiviral activity. In addition, it is not known how many ZAP molecules are required to bind RNA to mediate antiviral activity or if they have to be clustered in a specific way. While structural and mutagenesis studies of the RNA binding domain in ZAP have shown that it is a dimer that may have two RNA binding cavities within a large RNA binding cleft, how it binds CpG dinucleotides remains unknown (66, 67). To fully understand how specific CpGs mediate ZAP-dependent antiviral activity, it will be essential to understand how ZAP binds CpGs in specific RNA contexts and structures and the role its cofactors play in modulating its RNA binding activity.

It will be interesting to compare how CpGs inhibit HIV-1 to how they inhibit other RNA viruses and if they do so by targeting viral RNA for degradation, by inhibiting its translation, or through other mechanisms. CpGs directly or indirectly introduced into coding and noncoding regions of picornaviruses have shown that they can potently attenuate viral replication and create strains that protect animals from challenge with the wild-type virus (6–8, 10, 68). ZAP has recently been shown to be necessary for introduced CpGs to inhibit the picornavirus echovirus 7 (32). CpGs have also been shown to attenuate influenza A virus and to protect animals from lethal challenge by the wild-type virus (9). However, the molecular mechanism of attenuation remains unclear, and the CpGs could inhibit via ZAP, altered RNA splicing similar to what we have observed in HIV-1, or other mechanisms. This highlights both a challenge and an opportunity for introducing CpG dinucleotides to create live attenuated vaccines. The multiple mechanisms of action, as well as the position and context dependence of CpG-mediated viral inhibition, pose a challenge to determining the engineering principles for attenuating viruses with a predicted magnitude and mechanism. The opportunity is that CpGs can be used to attenuate viruses through multiple and potentially additive or synergistic mechanisms, which may enhance the utility of this approach.

## MATERIALS AND METHODS

### Sequence analysis of viral genomes.

The “analyze base composition” tool in MacVector (MacVector Inc.) was used to calculate the CpG and UpA observed/expected ratios for the viral sequences. The observed/expected ratio was calculated using the following formula: number of dinucleotide occurrences/(frequency of first nucleotide × frequency of second nucleotide), where the frequency of the base is the number of occurrences of the base divided by the total number of bases in the sequence. For the retroviral sequences, the sequence representing the packaged genome was used, i.e., the sequence encompassing the 5′ repeat (R) to the 3′ R.

### Synonymous site conservation analysis.

Synonymous site conservation was calculated with synplot2 (37) for codons in a *gag-pol-vif-vpr*-5′ *tat-vpu-env-nef* in-frame fusion. The 16-nt noncoding sequence between *tat* and *vpu* was deleted, the frameshift heptanucleotide was changed from UUUUUUA to UUUUUUAA to fuse *gag* and *pol*, and single-nucleotide insertions (N) or deletions were applied as necessary to fuse ORFs in frame. Sequences were translated into amino acid sequences, which were aligned with CLUSTAL W (69). The amino acid alignment was then converted back to a nucleotide alignment with tranalign from the EMBOSS package (70). The alignment was then mapped to the coordinates of a specific reference sequence (*viz*. EU541617) by removing alignment positions that contained a gap character in the reference sequence. After calculating the synonymous site conservation statistics with synplot2, they were mapped to EU541617 genome coordinates (i.e., by reinserting noncoding regions) for display (Fig. 2). The following GenBank sequences were used in the alignment: AB023804, AF321523, EU861977, JN944905, JX236669, M38429, AB287379, AY805330, FJ670516, JN944907, JX236670, AB485648, FJ771006, JN944941, JX236671, AB485649, FJ771007, JN944942, JX236672, AF004394, FJ771008, JN944943, JX236673, U26942, AF004885, FJ771009, JN944944, JX236676, U34603, AF005494, FJ771010, JN944945, JX236677, U34604, AF005496, GQ290462, JN944946, JX236678, U39362, AF077336, GU237072, JN944947, JX236679, U63632, AF082394, DQ676872, GU362882, JN944948, K03454, U88824, AF082395, DQ979025, JN106043, JX236668, M27323, AF286237, EU541617, KC156212, KC156213, KC156214, KC156215, KC156218, and KC156221. We also used the WARO, MCST, RHGA, STCOr1, and CH457 sequences, which have previously been described (71, 72). The z-scores for 9-, 15-, and 25-codon windows were as follows: 9-codon window, z ≥ 3.603808; 15-codon window, z ≥ 3.468872; 25-codon window, z ≥ 3.329161. These equate to a *P* value of 0.05, with a correction for multiple testing based on the number of independent (i.e., nonoverlapping) windows of length *x* codons in the 2,870-codon alignment, i.e., 0.05/(2,870/*x*). The scores correspond to the whole *x*-codon window centered on a given codon.

### Plasmids.

All primers were ordered from Eurofins, and the PCRs were performed with Phusion high-fidelity polymerase (New England Biolabs). Diagnostic restriction enzyme digestion and DNA sequencing (Eurofins Genomics) were used to ensure the correct identity of modified sequences inserted into plasmids. The pHIV-1_NL4-3_ constructs used in this study contain the provirus sequence from pHIV-1_NL4-3_ (73) cloned into pGL4.10 (Promega) (15). The sequences for HIV-1*_gag_*_22–378_DC, pHIV-1*_gag_*_22–654_CpG, pHIV-1*_gag_*_654–1188_CpG, pHIV-1*_gag_*_22–1188_CpG, pHIV-1*_gag_*_694–1204_CpG, pHIV*_env_*_611–1014_CpG, and pHIV-1*_pol_*_795–1386_CpG were synthesized by Life Technologies and cloned into pHIV-1_NL4-3_. CpG dinucleotides were introduced through synonymous mutations without altering cryptic splice sites predicted by the MaxEnt tool in Human Splicing Finder (74, 75) in pHIV-1*_gag_*_22–654_CpG, pHIV-1*_gag_*_654–1188_CpG, pHIV-1*_gag_*_22–1188_CpG, pHIV-1*_gag_*_694–1204_CpG, pHIV*_env_*_611–1014_CpG, and pHIV-1*_pol_*_795–1386_CpG. To generate pHIV-1 Gag-Venus, the SphI and XmaI fragment of pGag-Venus (43) was amplified via PCR and subcloned into pHIV-1_NL4-3_ in pGL4.10. pHIV-1 Gag-STOP-Venus was generated by PCR amplification of the SphI and XmaI sites in pGag-Venus using a primer that introduced the 3 stop codons, which was then subcloned into pHIV-1Gag-Venus. Hemagglutinin (HA)-*Renilla*, HA–ZAP-S, and HA–ZAP-L sequences were amplified by PCR and cloned into pcDNA3.1(+) (Invitrogen). pHIV-1*_gag_*_22–165_CM, pHIV-1*_gag_*_22–261_CM, pHIV-1*_gag_*_22–378_CM, HIV-1*_gag_*_22–378_CM–no-CpG, pHIV-1*_gag_*_22–378_CpG, pHIV-1-IRES-GFP, pHIV-1-IRES-*Renilla*, pHIV-1*env* 86–561CpG, pKHNYN-1, pGFP-FLAG, pGFP, and pVSV-G have been previously described (15, 31, 41, 42, 76, 77). Of note, pHIV-1*_gag_*_22–165_CM, pHIV-1*_gag_*_22–261_CM, and pHIV-1*_gag_*_22–378_CM were previously called pHIV-1 CM22–165, pHIV-1 CM22–261, and pHIV-1 CM22–378, respectively (15). pHIV-1*_gag_*_22–378_CM–no-CpG was previously called pHIV-1 CM22–378_lowCpG_, and pHIV-1*_gag_*_22–378_CpG was called pHIV-1 CpG22–378 (15). pHIV-1*_env_*_86–561_CpG was previously called pHIV-1_EnvCpG86–561_ (31).

### Cell culture and cell lines.

HeLa, TZM-bl, and HEK293T cells were cultured in Dulbecco’s modified Eagle medium (Gibco) supplemented with 10% fetal bovine serum (FBS) and 1% penicillin-streptomycin. All the cell lines were grown at 37°C in a humidified atmosphere with 5% CO_2_. For the production of ZAP and KHNYN knockout cell lines by CRISPR-Cas9, ZAP- and KHNYN-targeting guide sequences were inserted into a lentivirus-based CRISPR plasmid (LentiCRISPRv2-puro) from Addgene (no. 52961) (78). The CRISPR guide sequences were as follows: Luciferase-G1 (control), 5′-CTT TAC CGA CGC ACA TAT CG-3′; ZAP-ex4, 5′-TCT GGT AGA AGT TAT ATC TG-3′; ZAP-ex6, 5′-ACT TCC ATC TGC CTT ACC GG-3′; and KHNYN-ex3, 5′-GGG GGT GAG CGT CCT TCC GA-3. LentiCRISPR vectors encoding guide RNAs targeting luciferase or ZAP were produced in HEK293T cells seeded in 6-well plates using 10 μl polyethylenimine (PEI) with 0.5 μg pVSV-G (76), 1.0 μg pCMVΔR8.91 (79), and 1.0 μg LentiCRISPRv2 (78). Virus-containing supernatant was harvested 48 h after transfection, rendered cell free via filtration through 0.45-μm filters (Millipore), and used to transduce HeLa or HEK293T cells, followed by selection in 1 μg/ml puromycin (Sigma-Aldrich). ZAP CRISPR HeLa cell clones were generated by limiting dilution. The ZAP-ex6 and KHNYN-ex3 CRISPR clones have previously been described and were called ZAP-G1 CRISPR clone and KHNYN-G1 CRISPR clone B (31). Loss of ZAP protein expression was confirmed by Western blotting.

### Transfections.

HeLa and HEK293T cells were seeded in 6-well plates and transfected at a confluence of 70%. HeLa cells were transfected using TransIT-LT1 (Mirus) according to the manufacturer’s instructions at a ratio of 3 μl TransIT-LT1 to 1 μg DNA. HEK293T cells were transfected according to the manufacturer’s instructions using PEI (1 mg/ml; Sigma-Aldrich) at a ratio of 4 μl PEI to 1 μg DNA. For each transfection, 0.5 μg pHIV-1 and 0.5 μg pGFP, pGFP-FLAG, pKHNYN-1, pVSVG, pHA-ZAP-S, pHA-ZAP-L, or pHA-*Renilla* was used, for a total of 1 μg DNA. The transfection medium was replaced with fresh medium 6 h (HEK293T) or 24 h (HeLa) posttransfection. The cells were lysed 48 h posttransfection, and the medium was recovered. In experiments performed with type I interferon, 1,000 U/ml of IFN-I (Universal Type I Interferon; PBL Assay Science) was added to the cells upon medium change 6 h posttransfection. The medium was filtered through a 0.45-μm filter, and the virions were pelleted for 2 h at 20,000 × *g* through a 20% sucrose cushion in phosphate-buffered saline (PBS) solution.

### TZM-bl cell infectivity assay.

Supernatant was recovered 48 h posttransfection and filtered as previously described. TZM-bl cells were seeded at 70% confluence in 24-well plates and infected by overnight incubation with filtered virus stocks. Forty-eight hours postinfection, the cells were lysed, and the amount of infectious-virus production was measured by induction of β-galactosidase using the Galacto-Star system (Applied Biosystems) following the manufacturer’s instructions. β-Galactosidase activity was quantified as relative light units per second using a PerkinElmer luminometer.

### Analysis of protein expression by immunoblotting.

Approximately 48-h posttransfection, HeLa or HEK293T cells were lysed in radioimmunoprecipitation assay (RIPA) buffer (10 mM Tris-HCl, pH 7.5, 150 mM NaCl, 1 mM EDTA, 0.1% sodium dodecyl sulfate [SDS], 1% Triton X-100, 1% sodium deoxycholate). The medium was filtered through a 0.45-μm filter, and the virions were pelleted through a 20% sucrose cushion in PBS solution for 2 h at 20,000 × *g*. The pellet was resuspended in 2× loading buffer (60 mM Tris-HCl, pH 6.8, 10% β-mercaptoethanol, 10% glycerol, 2% SDS, 0.1% bromophenol blue). Cell lysates and virions were resolved by SDS-polyacrylamide gel electrophoresis and transferred to a nitrocellulose membrane. The antibodies used in the study were 1:50 anti-HIV-1 p24^Gag^ (mouse; NIH AIDS Reagent Program; catalog no. 1513) (80), 1:3,000 anti-HIV-1 gp160/120 rabbit (ADP421; Centralized Facility for AIDS Reagents [CFAR]), 1:1,000 anti-Hsp90 (sc7947; Santa Cruz Biotechnology), 1:5,000 β-actin (ac-15; Sigma), 1:5,000 anti-ZAP (Abcam; ab154680), 1:2,500 anti-HA (Biolegend; 901514), 1:2,500 anti-DYKDDDDK (rabbit; Cell Signaling; 14793), 1:1,000 anti-HIV-1 Nef (mouse; NIH AIDS Reagent Program; catalog no. 1539) (81). 1:10,000 Dylight 800-conjugated anti-mouse/rabbit secondary antibodies (Cell Signaling Technology; 5151S and 5257S), 1:5,000 anti-mouse IRDye 680RD (Li-CoR; 926-68070), 1:5,000 anti-rabbit horseradish peroxidase (HRP) (Cell Signaling Technology; 7074), and 1:5,000 anti-mouse HRP (Cell Signaling Technology; 7076). Bound primary antibodies were detected via Li-CoR infrared imaging (Li-CoR UK Ltd.) or using the Amersham ECL Prime Western blotting detection reagent (GE Life Sciences) for HRP-linked antibodies using an ImageQuant (LAS4000 Mini).

### Quantitative reverse transcription (qRT)-PCR.

HeLa cells were transfected at a confluence of 70% in a 6-well plate and after 48 h were washed with 1× PBS and lysed. The RNA was extracted using an RNeasy kit (Qiagen) following the manufacturer’s instructions. The supernatant was also collected and treated for 3 h at 37°C with RQ1 DNase (Invitrogen) to decrease plasmid DNA contamination. cDNA was synthesized using a high-capacity cDNA archive kit (Applied Biosystems), and 1 μg of RNA from virions was isolated using a QIAamp viral RNA minikit following the manufacturer’s instructions. Because carrier RNA was added to the lysis buffer, the total RNA isolated was quantified using a Qubit 3.0 fluorometer (ThermoFisher) and normalized so that 20 ng of RNA from each sample was reverse transcribed using the high-capacity cDNA archive kit (Applied Biosystems). Quantitative PCRs (qPCRs) were performed in triplicate with TaqMan universal PCR mix using an Applied Biosystems 7500 real-time PCR system. The HIV-1_NL4-3_ gRNA primers were GGCCAGGGAATTTTCTTCAGA/TTGTCTCTTCCCCAAACCTGA (forward/reverse), and the probe was 6-carboxyfluorescein (FAM)-ACCAGAGCCAACAGCCCCACCAGA-6-carboxytetramethylrhodamine (TAMRA). The HIV-1_NL4-3_ total RNA primers were TAACTAGGGAACCCACTGC/GCTAGAGATTTTCCACACTG (forward/reverse), and the probe was FAM-ACACAACAGACGGGCACACACTA-TAMRA. The absolute number of copies was determined using the slope of the standard curve at a qPCR efficiency between 95% and 105%.

### Analysis of HIV-1 splicing.

HeLa cells were transfected at a confluence of 70% in a 6-well plate and after ∼40 h were washed with 1× PBS and lysed. The RNA was extracted using an RNeasy kit (Qiagen) following the manufacturer’s instructions. The RNA samples were quantified using a Qubit 2.0 fluorometer (Life Technologies, Carlsbad, CA, USA), and RNA integrity was checked with RNA screen tape on an Agilent 2200 TapeStation (Agilent Technologies, Palo Alto, CA, USA). The multiplexed RNA-sequencing library was prepared using an Illumina TruSeq stranded mRNA library preparation kit following the manufacturer’s protocol. Sequencing libraries were validated using DNA analysis screen tape on an Agilent 2200 TapeStation and quantified by using a Qubit 2.0 fluorometer, as well as by quantitative PCR (Applied Biosystems, Carlsbad, CA, USA). The sequencing libraries were multiplexed and clustered on two flow cell lanes. After clustering, the flow cell was loaded on the Illumina HiSeq instrument according to the manufacturer’s instructions. The samples were sequenced, using a 2-by-150 paired-end (PE) high-output configuration, by Genewiz. Image analysis and base calling were conducted with HiSeq control software (HCS) on the HiSeq instrument. The raw sequence data (.bcl files) generated from the Illumina HiSeq were converted into fastq files and demultiplexed using the Illumina bcl2fastq program version 2.17. Adapter trimming was performed using BBDuk (https://jgi.doe.gov/data-andtools/bbtools/), and read pairs were merged with BBmerge (https://jgi.doe.gov/data-and-tools/bbtools/) in order to increase base call quality and generate long, whole-fragment reads. The reads were then aligned with the human genome (hg38) and the HIV_NL4-3_ genomic-RNA sequence simultaneously using Hisat2 (82). HIV-mapping junction-spanning reads were isolated using regtools (https://github.com/griffithlab/regtools) to allow per-junction read counting. To visualize junctions of interest, data from replicates were first merged using the Picard (http://broadinstitute.github.io/picard) MergeSamFiles function, followed by generation of sashimi plots using Gviz (83). The percentage of HIV-1 junction-spanning reads was calculated by dividing the number of reads for each junction by the total number of junction-spanning reads in the library.

### Statistical analysis.

Statistical significance was determined using unpaired two-tailed *t* tests calculated using Microsoft Excel software. Data are represented as means ± standard deviations. Significance was ascribed to *P* values of <0.05.

### Data availability.

The RNA-seq data are available in the NCBI Gene Expression Omnibus (GEO) under accession number GSE134159. The GenBank accession numbers for the HIV-1 genomic-RNA nucleotide sequences are as follows: HIV-1 (strain NL4-3), MN685337; HIV-1*_gag_*_22–165_CM, MN685338; HIV-1*_gag_*_22–261_CM, MN685339; HIV-1*_gag_*_22–378_CM, MN685340; HIV-1*_gag_*_22–378_CM–no-CpG, MN685341; HIV-1*_gag_*_22–378_CpG, MN685342; HIV-1*_gag_*_22–378_DC, MN685343; HIV-1*_gag_*_22–378_CM–5nt-CpG, MN685344; HIV-1*_gag_*_22–651_CpG, MN685345; HIV-1*_gag_*_660-1185_CpG, MN685346; HIV-1*_gag_*_22–1185_CpG, MN685347; HIV-1*_gag_*_694–1206_CpG, MN685348; HIV-1*_pol_*_795–1386_CpG, MN685349; HIV-1*_env_*_86–561_CpG, MN685350; HIV-1*_env_*_611–1014_CpG, MN685351; HIV-1*_env_*_86–1014_CpG, MN685352.

## Supplementary Material

Supplemental file 1

Supplemental file 2

Supplemental file 3
